# Understanding the Cholera Epidemic, Haiti

**DOI:** 10.3201/eid1711.110981

**Published:** 2011-11

**Authors:** Sher Bahadur Pun

**Affiliations:** Author affiliation: Sukraraj Tropical and Infectious Disease Hospital, Teku, Nepal

**Keywords:** cholera, epidemic, bacteria, outbreak, Haiti, epidemiology, Vibrio cholerae

## Abstract

After the devastating outbreak of cholera in Haiti in mid-October 2010, several hypotheses have emerged regarding the origin of the outbreak. Some articles and media reports pointed to the United Nations peacekeepers from Nepal as the source. Piarroux et al. drew a similar conclusion from their epidemiologic study (*1*). Nepal did experience an outbreak of cholera during August–October 2010, in which 72 cases of infection with *Vibrio cholerae* O1, serotype Ogawa, were confirmed, mostly among young adult males. The cases peaked from mid-September to early October (Figure; Figure A1), and no deaths occurred. Despite this similarity in timing, I believe several points need to be considered before a firm conclusion is reached.

Cholera strains isolated in Haiti were genetically most similar to strains detected in Bangladesh in 2002 and 2008; thus, cholera was most likely introduced into Haiti from southern Asia (*2*). Despite the genetic similarity in the strains, no attempt was made by the researchers to ascertain and rule out the source of the outbreak in Bangladeshi policemen stationed at Mirebalais between September and October 2010. Another, although less likely, source for the introduction of cholera into Haiti could have been travelers or relief workers who may have recently been to southern Asia. Most relief workers probably come from countries without endemic cholera, but they cannot definitely be ruled out as a source of cholera in Haiti. For example, in industrialized countries, cholera has been detected among travelers, albeit in smaller numbers, returning home from cholera-endemic areas (*3**,**4*). However, Piarroux et al. offered no information about travelers or relief workers or whether they had been screened for *V. cholerae* infection before coming to Haiti (*1*). Of note, the United Nations reported that none of the Nepalese peacekeepers was found to be positive for the strain in Haiti (*5*); hence, other possible explanations for the origin of the outbreak simply cannot be overlooked.

**Figure:**
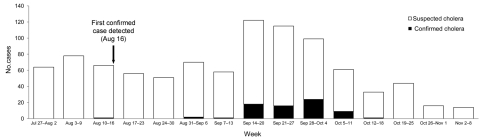
Patients with confirmed and suspected cases of cholera admitted to Sukraraj Tropical and Infectious Disease Hospital, by week, Katmandu, Nepal, July–November 2010. Case definitions: suspected cholera, acute watery diarrhea, with or without vomiting, in a child >5 years of age; confirmed cholera, isolation of *Vibrio cholerae* O1 or O139 from feces of any patient with diarrhea.

## Figures and Tables

**Figure A1:**
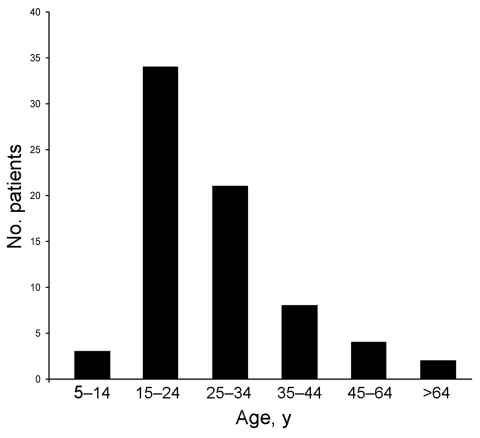
Patients with confirmed cases of cholera admitted to Sukraraj Tropical and Infectious Disease Hospital, by age group, Katmandu, Nepal, July–November 2010.
