# Supplementary material for: Understanding the Cholera Epidemic, Haiti
Source: Emerg Infect Dis. 2011 Nov;17(11):2178–9. doi: 10.3201/eid1711.110981 (PMC3310591; doi:10.3201/eid1711.110981)
Supplement: Related texts

# Reply: Understanding the Cholera Epidemic, Haiti

**DOI:** 10.3201/eid1711.111318

**Authors:** Renaud Piarroux, Robert Barrais, Benoît Faucher, Rachel Haus, Martine Piarroux, Jean Gaudart, Roc Magloire, Didier Raoult

**Affiliations:** Author affiliations: Université de la Méditerranée, Marseilles, France (R. Piarroux, B. Faucher, J. Gaudart, D. Raoult);; Ministère de la Santé Publique et de la Population, Port-au-Prince, Haiti (R. Barrais, R. Magloire);; Service de Santé des Armées, Paris, France (R. Haus);; Université de Franche-Comté, Besançon, France (M. Piarroux)

**In Response:** We read with great interest the letter by Pun, which suggests that Bangladeshi policemen in Mirebalais could have introduced cholera into Haiti (*1*). However, we want to emphasize that the first Haitian cholera case occurred in Meille, just next to the Nepalese military camp—not in Mirebalais or Hinche, where Bangladeshi policemen served. The location of the first case was stated in our article (*2*) and confirmed by the United Nations (UN) panel of experts on the cholera outbreak in Haiti (*3*). The UN panel also reported that major sanitation deficiencies likely resulted in contamination of a stream flowing within a few meters of the Nepalese camp. No other humanitarian forces were working in the small hamlet of Meille.

As acknowledged by Pun, Nepalese soldiers left for Haiti just when a cholera epidemic was raging in their country. According to the UN panel report, “a careful analysis of the MLVA [multilocus variable-number tandem-repeat analysis] results and the *ctxB* gene indicated that the strains isolated in Haiti and Nepal during 2009 were a perfect match.” Nepalese strains had been made available to the UN Panel from the International Vaccine Institute in Seoul, South Korea (*3*).

Referring to UN press conferences, Pun stated that “none of the Nepalese peacekeepers was found to be positive for the [*V. cholerae*] strain in Haiti.” However, it should be remembered that no testing of the soldiers was performed. Although the UN panel reported that “no cases of severe diarrhea and dehydration occurred among MINUSTAH [United Nations Stabilization Mission in Haiti] personnel during this period,” the panel provided no information concerning mild or moderate diarrhea.

Overall, evidence overwhelmingly supports the conclusion that the UN military camp in Meille was the source of the Haitian cholera epidemic. The person who brought cholera into Haiti could not be identified because of the lack of an early, independent investigation in the camp.

## References

[1] Pun SB. Understanding the cholera epidemic, Haiti [letter]. Emerg Infect Dis. 2011;17:2178–9. 10.3201/eid1711.11098122204043PMC3310591

[2] Piarroux R, Barrais R, Faucher B, Haus R, Piarroux M, Gaudart J, Understanding the cholera epidemic, Haiti. Emerg Infect Dis. 2011;17:1161–8. 10.3201/eid1707.11005921762567PMC3381400

[3] Cravioto A, Lanata CF, Lantagne DS, Nair GB. Final report of the independent panel of experts on the cholera outbreak in Haiti [cited 2011Sep 2]. http://www.un.org/News/dh/infocus/haiti/UN-cholera-report-final.pdf
